# Taking the Case*Read before the Kansas State Homœopathic Society, May, 1897.

**Published:** 1897-10

**Authors:** W. A. Yingling

**Affiliations:** Emporia, Kansas


### TAKING THE CASE.*

W. A. Yingling, M. D., Emporia, Kansas.

The art of Homœopathy is the application of potentized medicine to a sick condition as expressed in the totality of symptoms. The artist of Homœopathy is the one who can individualize the sick condition and interpret the language of symptomatology, both of the patient and the drug. The true artist must have the ability to acquire a true picture of each individual case as expressed in the totality of symptoms, and the acumen to select a remedy having a true likeness to the totality. He must analyze the symptomatic condition of the patient on the one hand, and synthetize the pathopoiesis of the remedy on the other. For a proper synthesis of the remedy a thorough analysis of the patient is not only important but necessary. Hence, "Taking the Case," which has become a technical phrase in the literature of Homœopathy, is the *sine qua non* of a curative prescription. The leading pre-

* Read before the Kansas State Homœopathic Society, May, 1897.

scribers unite in asserting that "Taking the Case" is the physician's most arduous and laborious work, and usually the cause of failure.

Symptoms are but the language of Nature speaking to the intelligent physician. Every diseased condition is expressed by certain signs, and when the condition is the same the symptoms will be the same. The variation of symptoms indicates a variation in the condition. The peculiar or characteristic symptoms in a given case point to the individual peculiarities of the patient. The language of Nature expressing a diseased condition varies, even in the same pathological state, simply because the individuality of the patient's vital force has been differently attacked or affected, or the vital force has been differently influenced by some extraneous or internal power. This variation in the symptomatology of any given pathological condition is the most unerring guide in the selection of the curative homœopathic remedy.

There are two classes of symptoms, the pathological and the idiosyncratic. The literal meaning of this term idiosyncratic, which is composed of three Greek words, is, with peculiar temperament, hence the idiosyncratic symptoms are those peculiar to the patient's temperament, those arising out of the patient's individuality, and are in contradistinction to those arising out of the pathological condition. The pathological class of symptoms has reference to the diseased condition, and are only of value for diagnosis or prognosis. The idiosyncratic symptoms point to the individuality of the patient, and are only of value in the selection of the homœopathic remedy. The pathological symptoms, which point unerringly to the name of the disease, are seldom if ever of any value in the selection of a curative remedy. Likewise, the idiosyncratic symptoms are of no value to the pathologist. Yet both classes are of the highest value in their sphere of usefulness to the homœopathic physician. The homœopathician must be both a pathologist and a prescriber. He must have the keenest sense of discrimination and of judgment.

The two classes of symptoms require on the part of the physician two kinds of knowledge. He must be versed in pathology, all that pertains to diagnosis, and in symptomatology, all that pertains to the homœopathic materia medica. If he does not know pathology he will not be able to question the patient, or to understand what he sees, so as properly to diagnose and prognose his case. If he does not know his materia medica he will not properly understand how to get those symptoms, or will not know them when he sees them, upon which he must almost exclusively base his prescription. These idiosyncratic symptoms, those peculiar to the individual patient, are those to which Hahnemann refers in Section 153, of his *Organon of the Healing Art.*

In taking the case one of the essentials is to get the "totality of symptoms," especially the totality of idiosyncratic symptoms. This totality does not alone refer to the aggregate of symptoms, but particularly to the totality or completeness of each symptom. Each complete symptom has its 1, location; 2, sensation; 3, modalities, conditions of aggravation and amelioration; and 4, concomitants. In any given disease one *complete* symptom is worth a dozen incomplete or fractional symptoms in the selection of the homœopathic curative remedy. Hahnemann says in Section 153: "The more general and *indefinite* [that is, the incomplete] symptoms, such as want of appetite, headache, weakness, restless sleep, distress, etc., unless more clearly defined, deserve but little notice on account of their vagueness, and also generalities of this kind are common to every disease, and almost to every drug."

This is not a license to single out a part of the diseased condition, or a part of the symptoms, as a basis for a prescription, but indicates that the prescriber needlessly worries his brain and wastes his time in endeavoring to cover the totality of symptoms in the aggregate when he has only parts of symptoms, or fractional symptoms, with which to deal. He must endeavor to get *all* the symptoms of a case, and each symptom in its completeness or its totality. He need give but little attention to those symptoms in selecting a remedy he is unable by judicious questioning to complete.

The physician must ever keep before his mind these four questions in connection with each separate symptom: 1, Where is it? (the location); 2, What is it? (the sensation); 3, What affects it? (the conditions of aggravation and amelioration); 4, What occurs with it? (the concomitants). If he cannot elicit satisfactory answers to at least the first three of these questions his record is incomplete and his prescription will be uncertain.

It has been asserted from the time of Hahnemann that when the case has been properly taken more than half of the physician's labor has been accomplished. This is literally true. The hardest, the most laborious, the most important duty of the homœopathic physician is "taking the case." When the case has been fully taken the selection of the homœopathic curative remedy is easy. When thus taken that matchless of homœopathic books, Bœnninghausen's *Pocket Book*, opens up the intricacies of the ponderous materia medica to the busy doctor with speed and accuracy. By its aid the true remedy can be ascertained in less time than many physicians lose in debating uncertainties or in halting between two opinions, unable to decide what to do.

One of the "dont's" of taking the case is never to ask a leading question. This is *most important*, and should be conscientiously observed when possible, but unfortunately it is not universally applicable. There are patients and patients, some of whom understand themselves and some do not, and the latter class is not composed exclusively of the uneducated. I have known college graduates to be, seemingly, incapable of distinguishing the pain inflicted by a keen-edged knife or a blunt awl. To this class, whether educated or uneducated, a pain is a pain, and all pain is the same, no matter what its cause or its character. One fair damoselle, a graduate, in relating her symptoms, said she had a pain so much of the time in her "stomach." To her a pain was only a pain, and in reply to the question, "Where is it?" she placed her finger on the region of the left ovary.

Leading questions should be the last resort, and then they should be merely suggestive by analogy, and must always be verified by cross-examination and negative questioning. A symptom elicited by direct questioning with the answer "yes" is of but little value, because it is uncertain, unless verified by further examination. Some persons do not realize the importance of specific and exact location, sensation, and modality, and because of this lack of knowledge will answer "yes" to almost any question propounded. This does not arise from a purpose to deceive, but from an inability to conceive. The physician must be observing and wary, and govern his examination by the peculiar mental condition and grasp of the case in hand.

The true way to take a case is to ask the patient to state fully his complaints, and when he has completed his story to ask him to give particulars of each symptom as to its location, sensation, conditions of aggravation and amelioration, and the concomitants, noticing carefully the more marked, prominent, and peculiar symptoms, for upon these particularly the prescription must be based. It is very essential that the peculiarities of the patient's symptoms should be fully covered by the peculiarities of the selected remedy. Not only so, but the value of symptoms should correspond—that is, a very marked and prominent symptom of the patient should be equally marked and prominent in the remedy. By bearing this in mind during the examination of a patient the selection of the simillimum becomes more easy. And right here is the key to the use of a good repertory. The repertory is a sealed book to some physicians simply because they have not studied Hahnemann's *Organon* with assiduity and attention, so as to comprehend the distinction between symptoms and the manner of taking the case. In fact, I might parenthetically remark, all our failures as homœopathic physicians arise from our ignorance of the *Organon of the Healing Art* and the *Chronic Diseases*. The more we know of Hahnemann's writings the better prescribers we become.

Finally it is requisite that all the symptoms of a given case should be written down at the time of the examination by the physician. No mind can carry the totality of symptoms of even an easy case, and at the same time weigh the evidence for and against a number of possible remedies and successfully select one remedy to cover the totality. Some modality, sensation, or condition essential in the final decision will be overlooked when the record is not made in writing. It is a matter of fact that all our best prescribers, both of the past and the present, write in full each case.

This plan is not so laborious as it may seem, and it does not, very frequently, consume as much time as the uncertainty and requestioning of those who fail to write. Every word the patient says need not be written, only the symptoms briefly stated, yet in such a manner as to contain all the facts and peculiarities. Very often the patient may require ten minutes to clearly state a symptom that may be expressed in a dozen words. Any physician can easily accustom himself to a few abbreviations that will greatly aid him in taking a case, and with a minimum of labor.

The foregoing should be carefully studied and practiced. The Editor makes it his rule to note the statements of the patient upon specially-prepared blanks. The remedy given and the date are noted also, and the record filed away for future use.—Ed.

